# The Cancer That Vanished: A Case of Tuberculous Spondylitis Mimicking Metastatic Disease in a High-Stakes Diagnosis

**DOI:** 10.7759/cureus.96551

**Published:** 2025-11-11

**Authors:** Dristy Chowdhury, Adibul H Chowdhury, Monija Islam Diba, Denis D Taiwo

**Affiliations:** 1 Internal Medicine, Medway NHS Foundation Trust, Gillingham, GBR; 2 Acute Medicine, Medway NHS Foundation Trust, Gillingham, GBR

**Keywords:** diagnostic challenge, metastatic mimic, pott’s disease, radiotherapy complication, spinal tuberculosis

## Abstract

Skeletal tuberculosis remains a great clinical mimicker, often resembling metastatic malignancy on imaging. Prompt and accurate diagnosis is critical to prevent irreversible neurological damage and iatrogenic harm.

We describe a diagnostically challenging case involving a 39-year-old previously healthy woman from Zimbabwe who presented with subacute thoracic back pain and bilateral lower limb sensory changes. MRI demonstrated destructive vertebral lesions with spinal cord compression and widespread lymphadenopathy, strongly suggestive of metastatic disease. Further imaging, including contrast-enhanced CT and positron emission tomography (PET)-CT, supported the metastatic pattern but failed to identify a primary malignancy. The patient underwent spinal radiotherapy for presumed metastatic cord compression without clinical improvement.

A subsequent CT-guided biopsy revealed caseating granulomatous inflammation and acid-fast bacilli, confirming spinal tuberculosis. Following initiation of standard anti-tubercular therapy, the patient showed marked clinical improvement, including complete resolution of pain and partial neurological recovery. Unfortunately, some sensory impairment persisted due to radiation-induced myelitis - an avoidable complication of premature treatment.

This case signifies the importance of maintaining a high index of suspicion for spinal tuberculosis, especially in patients from endemic regions, even in the absence of classic systemic features. It is especially relevant to acute medicine, where clinicians are often the first to assess patients with spinal pain and red flag neurological signs. It highlights the need for histological confirmation before initiating oncologic therapies in uncertain diagnoses. Early tissue diagnosis, guided by WHO and national TB protocols, is essential to avoid misdiagnosis and ensure appropriate management.

## Introduction

Tuberculosis (TB) continues to be a major health challenge worldwide. While most cases (85-90%) affect the lungs, a significant minority (10-15%) occur outside the lungs, involving areas such as lymph nodes, the gastrointestinal and genitourinary tracts, skin, and the musculoskeletal system [1,2]. Among these extra-pulmonary forms, spinal TB (also known as Pott's disease) is the most common, representing nearly half of all osteoarticular TB cases [3].

Every year, an estimated 11 million people are affected by TB globally, and around 150,000 new cases of spinal TB are reported [4]. The majority of these - about two-thirds - come from high-burden regions such as South Asia, China, Nigeria, Indonesia, and South Africa [5]. In higher-income countries, spinal TB is less common, but diagnosis can still be complicated by factors such as HIV-related immunodeficiency and the growing problem of drug-resistant TB [6].

Spinal TB may involve vertebral bodies either contiguously or in a skip-lesion pattern. The most common way spinal TB presents is with persistent back pain, usually developing slowly and often without the typical “textbook” TB symptoms such as fever, weight loss, or night sweats [7]. On scans and clinical assessment, spinal TB can easily look like other serious conditions - including metastatic cancer, primary spinal tumors, or bacterial spinal infections - making it a real diagnostic challenge [8]. Other differential diagnoses to consider include brucellosis, fungal infections, degenerative spinal disease, and inflammatory conditions such as ankylosing spondylitis, which may also present with similar radiological or clinical features. Because of this overlap, patients often go through multiple investigations, ranging from blood tests and advanced imaging to CT-guided biopsies, before the true diagnosis becomes clear [9]. Despite these tools, the average time between symptom onset and definitive diagnosis ranges from four to six months [10].

Immunosuppression, particularly due to HIV, and vitamin D deficiency have been strongly associated with increased susceptibility to spinal TB, owing to their effects on host immunity and the containment of *Mycobacterium tuberculosis* (MTB) infection [11,12]. People living with HIV often develop atypical or widespread forms of TB. This occurs because HIV weakens cell-mediated immunity, which is vital for controlling latent infection. Vitamin D deficiency has a similar impact on the immune system. It reduces macrophage activity and the production of antimicrobial peptides that help contain MTB. Both factors increase susceptibility to spinal TB and can alter disease presentation, treatment response, and overall prognosis. When identified and treated early with standard anti-tubercular therapy (ATT), most spinal TB cases achieve complete clinical and radiological healing, with recent meta-analyses reporting success rates of approximately 90% [13]. However, when diagnosis and treatment are delayed, spinal TB can cause serious complications such as collapse of the affected vertebrae, deformity of the spine (kyphosis), and even permanent nerve damage leading to neurological problems [14].

This case was previously presented as a poster at the Society for Acute Medicine Conference 2025, held in September 2025 in Manchester, United Kingdom, where it was selected for presentation based on the abstract.

## Case presentation

A 39-year-old woman, originally from Zimbabwe, presented to us in early 2025 with about one month of history of progressive mid-back pain associated with numbness below the umbilicus. She was vaccinated with the BCG vaccine in her childhood. She moved to the UK in 2022. Until recently, she had been living a full and active life, working as a carer in the UK since 2022, fully independent, and with no known medical issues.

Her symptoms started gradually in December with localized thoracic back pain, followed by the onset of sensory disturbances in her lower abdomen and legs. Within just four weeks, these symptoms progressed to lower limb weakness, leading to significant functional decline. She became dependent on others for all her daily activities and required a walking frame for mobility. On systemic review, she did not experience fever, cough, shortness of breath, or weight loss.

On neurological examination, muscle strength in the lower limbs was reduced to Medical Research Council (MRC) grade 3/5 bilaterally. Fine touch sensation was diminished below the umbilicus, muscle tone was reduced, and muscle bulk was preserved. All deep tendon reflexes were intact. She was unable to stand without support, so the gait could not be assessed. There were no signs of bowel or bladder dysfunction, and saddle anesthesia was absent. Systemic and general examinations were otherwise unremarkable.

**Figure 1:**
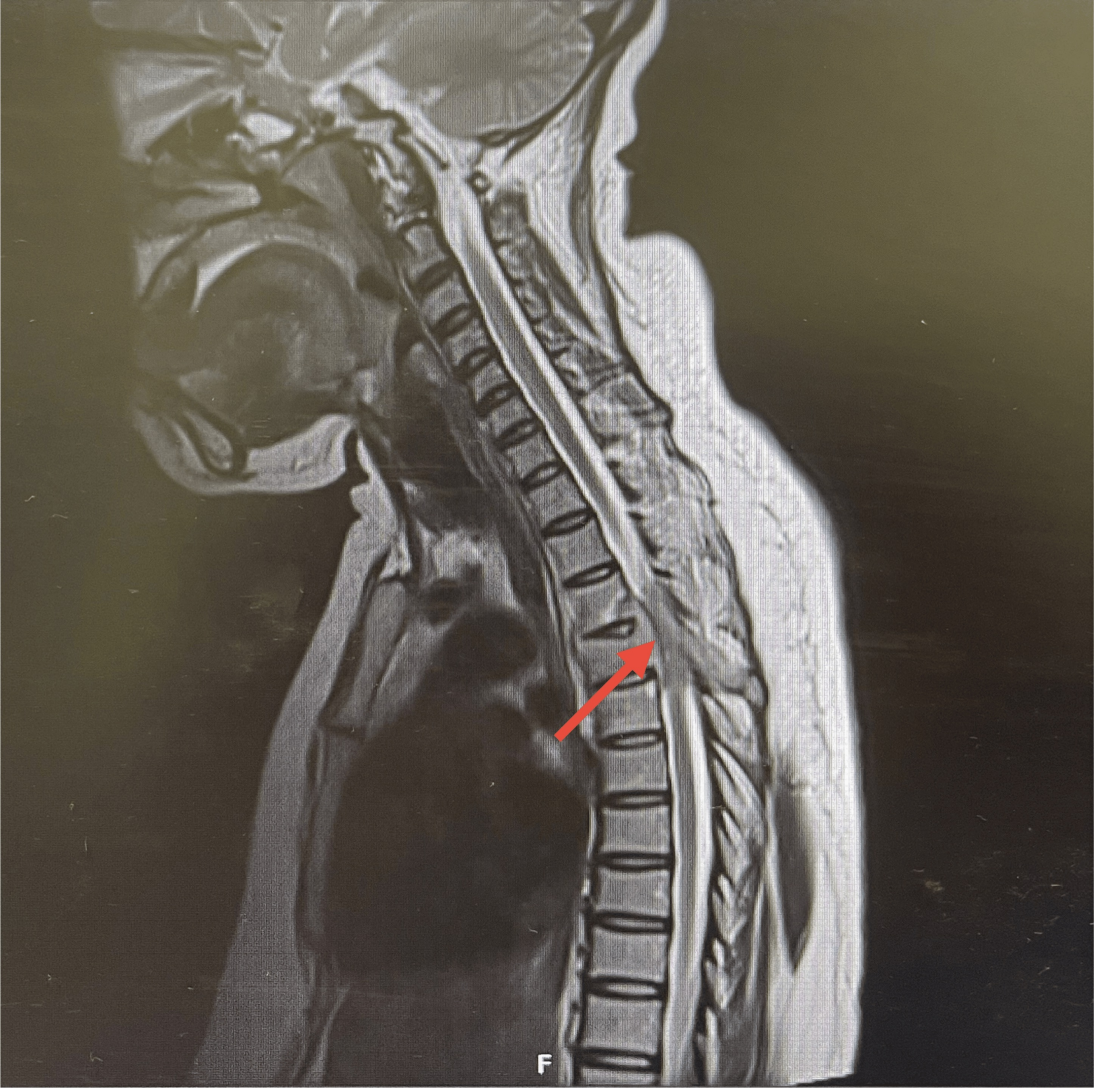
Pre-treatment MRI showing destructive changes involving T3-T5 vertebral bodies with significant T4 spinal cord compression (arrow).

**Figure 2:**
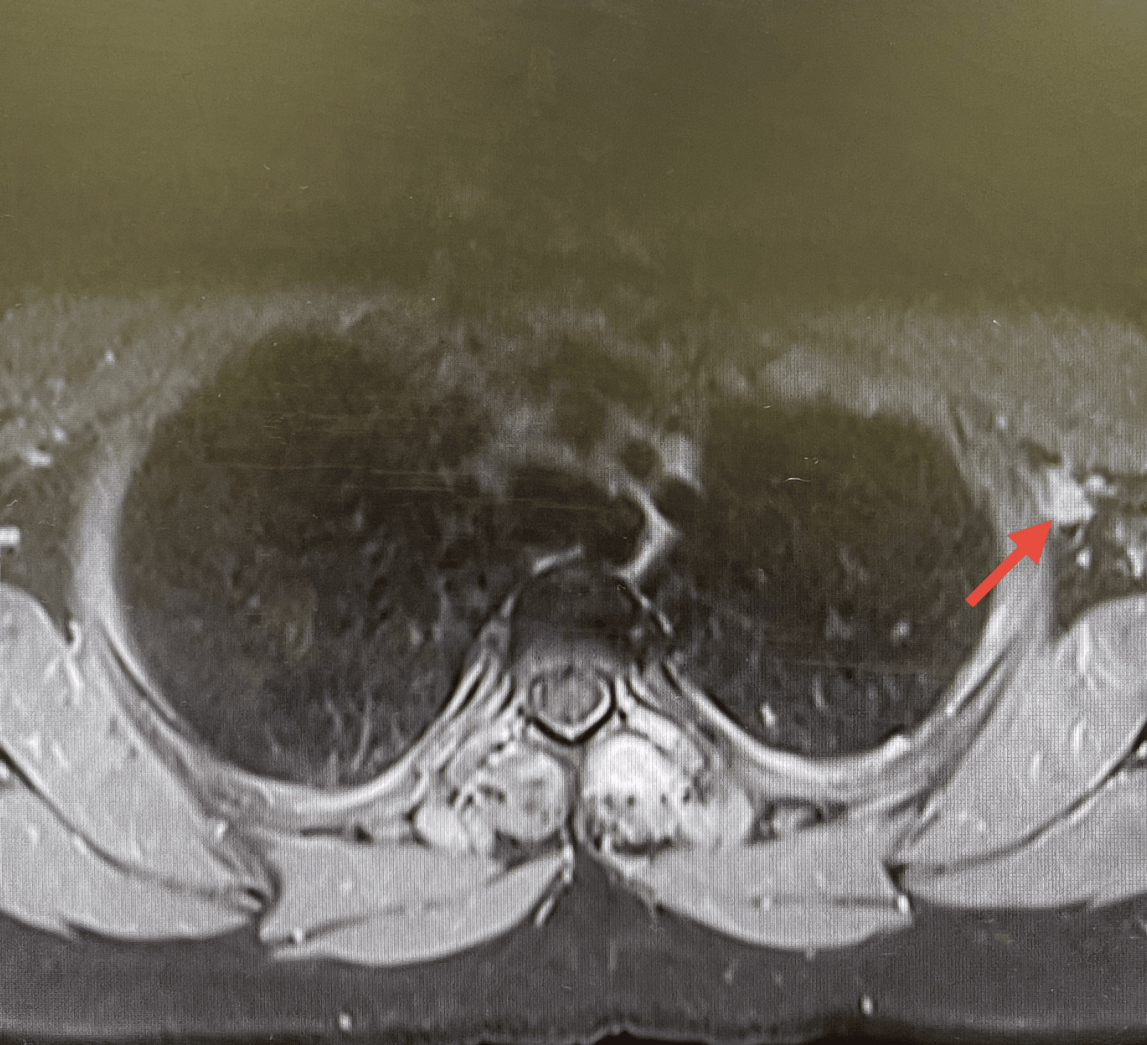
MRI showing enlarged left axillary lymph node (arrow), initially raising suspicion of metastatic disease.

**Figure 3:**
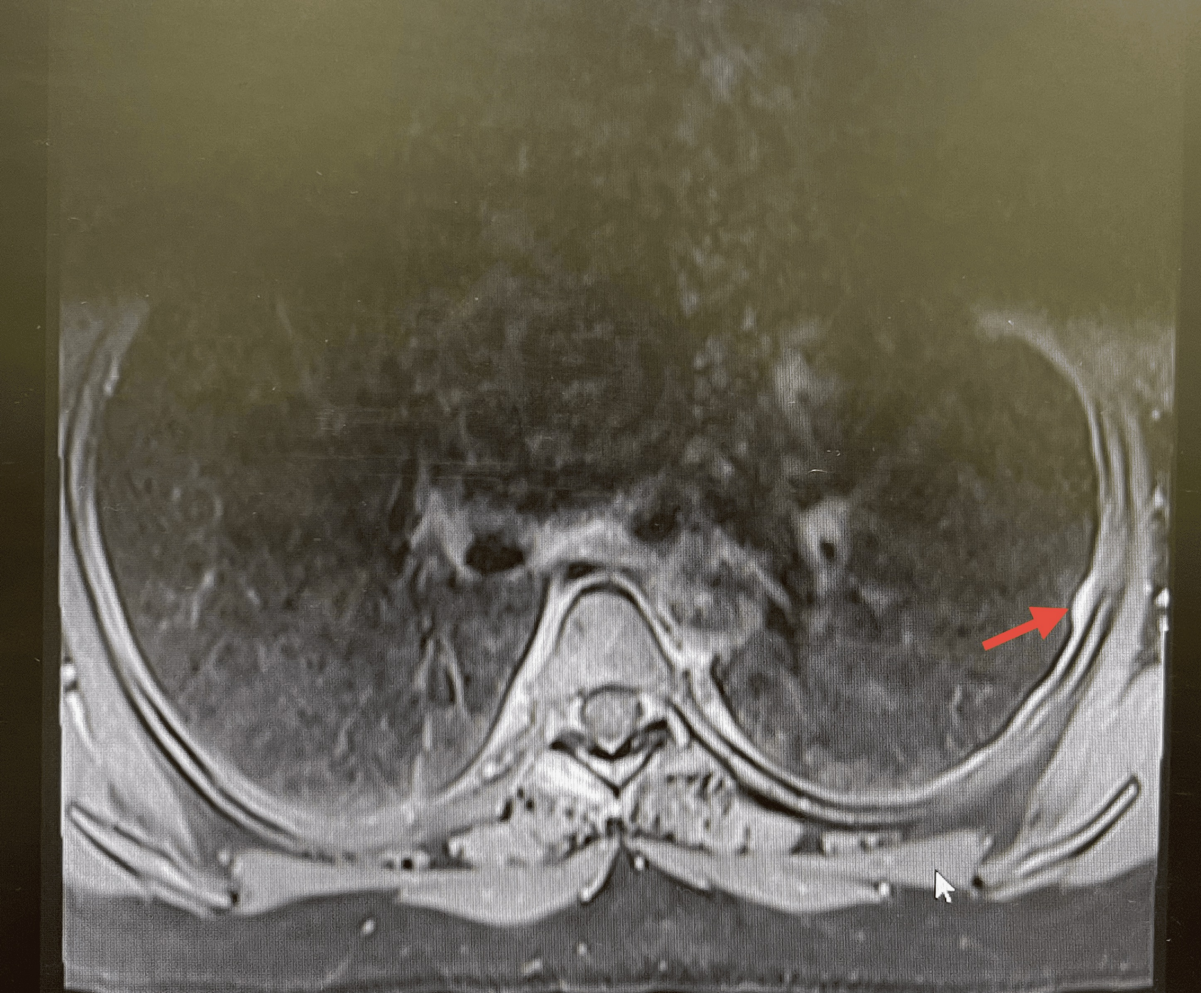
MRI showing focal left-sided pleural thickening (arrow), contributing to initial suspicion of metastatic spread.

Initial blood investigations, including complete blood count (hemoglobin, leukocyte, lymphocyte, neutrophil, and platelet counts), renal function, and inflammatory markers (white blood cell count, were within normal limits. Given the presence of red flag features, specifically progressive neurological deficits, an urgent MRI of the whole spine was performed. Imaging revealed destructive changes involving the T3-T5 vertebral bodies, with significant T4 spinal cord compression (Figure 1). Further findings include enlarged left axillary (Figure 2) and internal mammary lymph nodes. There was also left-sided focal pleural thickening (Figure 3). These findings gave rise to further strong suspicion towards metastatic malignancy, with a presumed breast primary.

**Figure 4:**
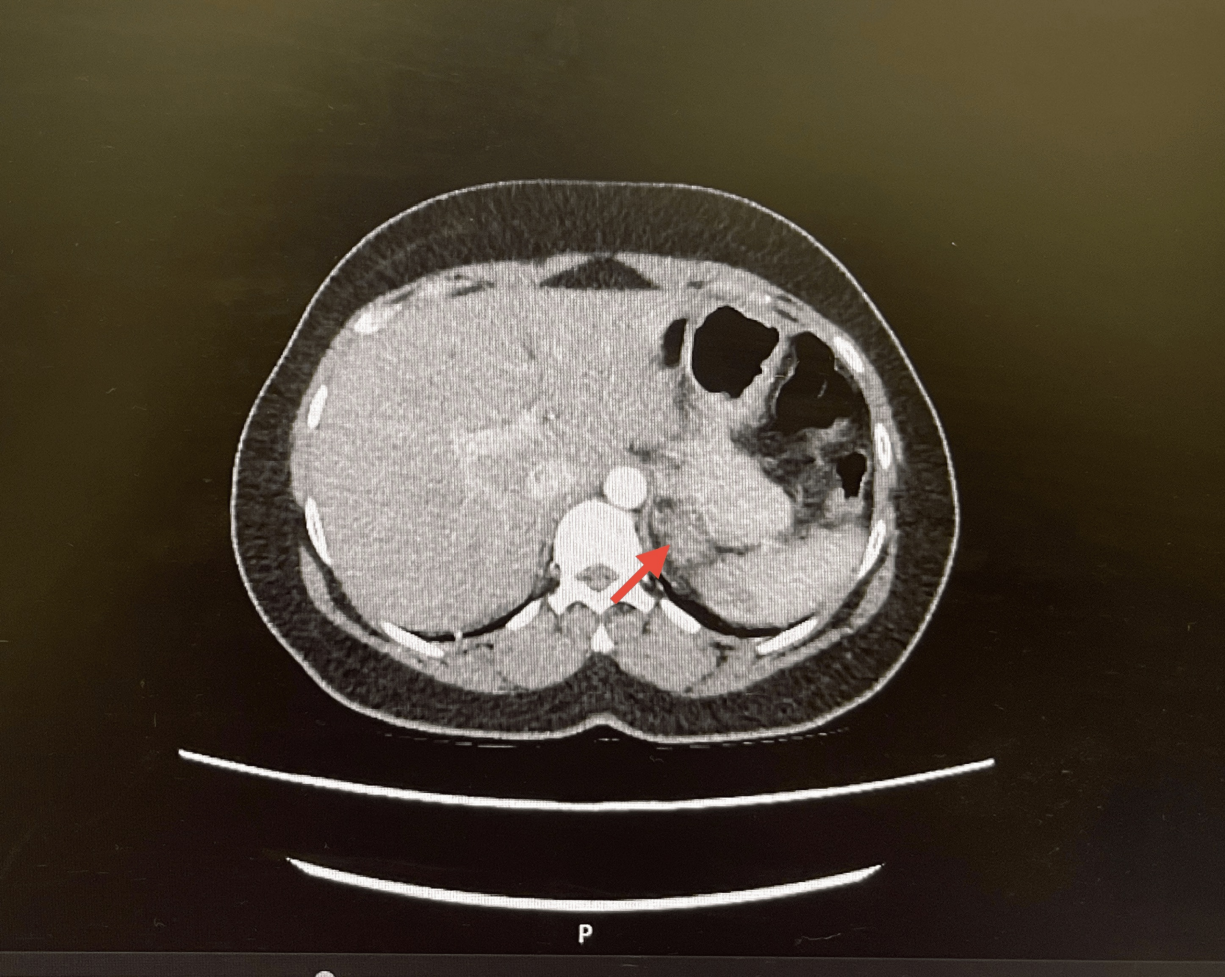
Contrast-enhanced CT scan showing left adrenal nodule (arrow), initially interpreted as a possible metastatic focus.

The next step was to conduct contrast-enhanced CT scans of the thorax, abdomen, and pelvis to seek the primary source, which failed to demonstrate a definitive primary lesion. However, there was evidence of additional suspicious features, which included a left adrenal nodule (Figure 4) and the presence of lytic lesions in the thoracic vertebrae, chest wall, and left iliac bone. All of this further supported a working diagnosis of disseminated metastatic disease.

Breast ultrasonography showed no suspicious masses. We also considered multiple myeloma, which was subsequently excluded through normal serum and urine protein electrophoresis. The case was discussed in several multidisciplinary team (MDT) meetings, including referral to a tertiary neurosurgical center for possible spinal decompression. However, they decided not to operate because her neurological status was stable.

**Figure 5:**
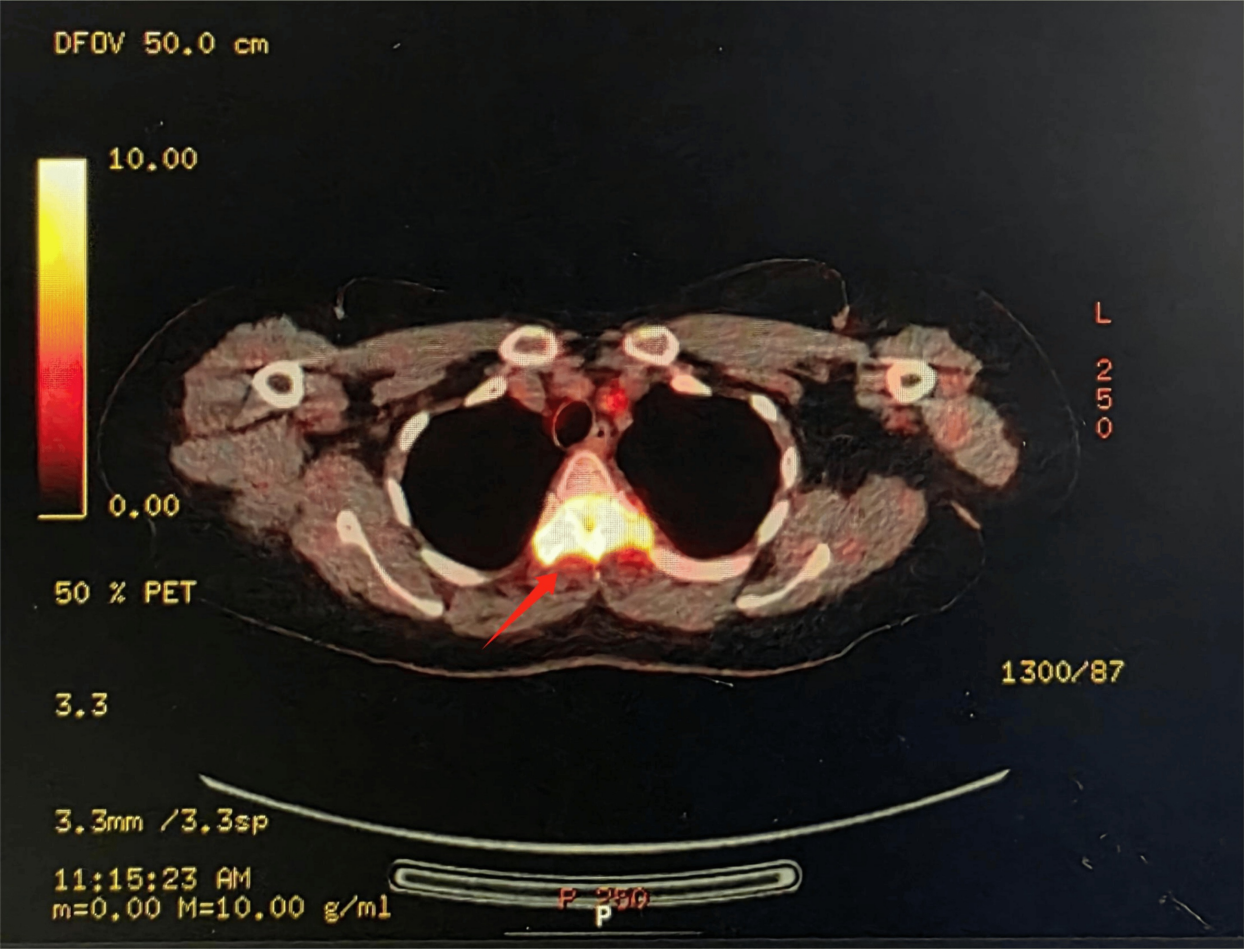
Positron emission tomography (PET)-CT showing hypermetabolic uptake at T4 vertebra (arrow), consistent with active inflammatory or neoplastic process.

A positron emission tomography (PET)-CT scan was performed and showed hypermetabolic lesions (Figure 5) in the skeletal and soft tissue sites, but again failed to reveal a primary malignancy. Given the diagnostic uncertainty and continued clinical decline, a CT-guided biopsy of the T4 vertebral lesion was undertaken, as recommended by the MDT.

While the patient underwent radiotherapy through the oncology team due to cord compression, there was no symptomatic improvement. Two weeks later, histopathological analysis of the spinal tissue surprisingly revealed caseating granulomatous inflammation consistent with MTB infection. The presence of multiple acid-fast bacilli (AFB) further strengthens the diagnosis of TB rather than malignancy.

**Figure 6:**
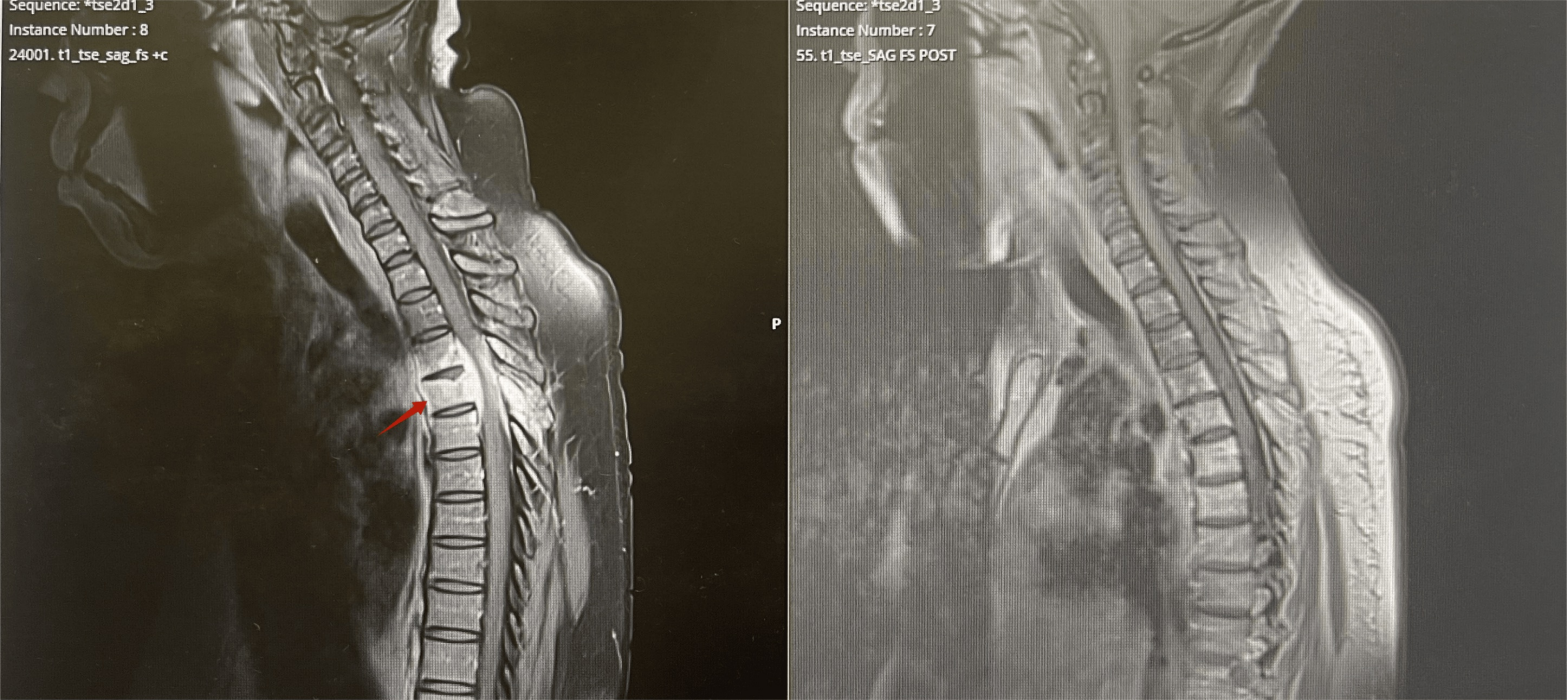
MRI of the spine before (left) and two months after (right) initiation of anti-tubercular therapy. Post-treatment image shows marked resolution of paravertebral enhancement and improved spinal cord decompression compared to pre-treatment MRI (arrow).

**Figure 7:**
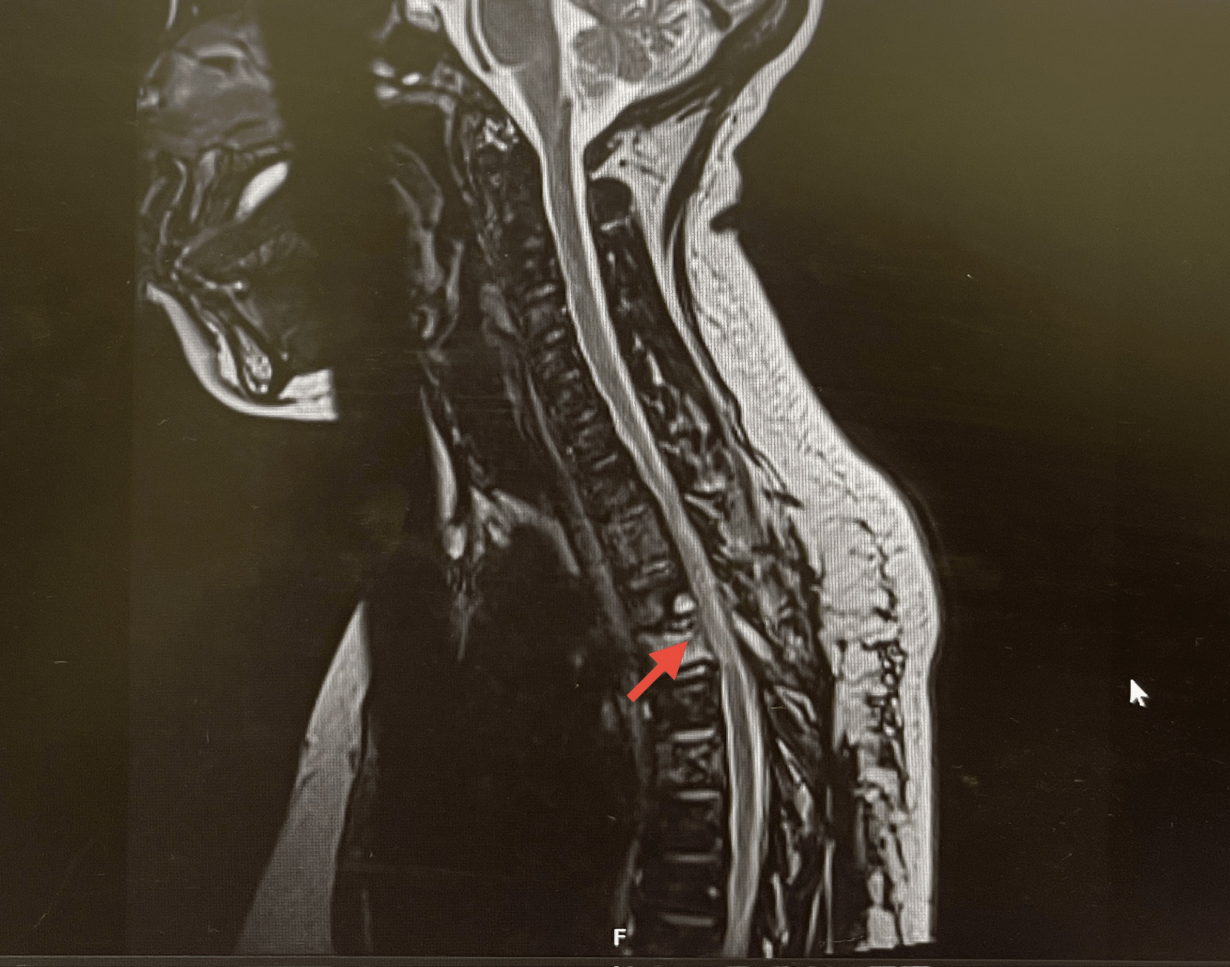
Follow-up MRI demonstrating T4-T5 hyperintense signal (arrow) consistent with radiation-induced myelitis.

After confirming spinal TB, the patient underwent full pre-treatment screening, including blood-borne viruses such as HIV, which returned negative. She was also noted to have a vitamin D deficiency, which was addressed accordingly. Later on, she was started on standard quadruple anti-TB therapy (isoniazid, rifampicin, pyrazinamide, and ethambutol). Her clinical response to the treatment was remarkable. Within weeks, her back pain and sensory deficit greatly improved. The improvement was evident in the repeat MRI as well (Figure 6). She gradually regained the ability to walk independently, which markedly improved her quality of life. Despite these positive changes, some degree of sensory impairment in the lower limbs remained. This suggests that continued rehabilitation may be necessary. Additionally, ongoing neurological monitoring would help to investigate the reason for the persistent sensory deficit that persisted. A repeat MRI of the spine was done, which shows changes likely due to radiation-induced myelitis due to the earlier radiotherapy (Figure 7).

## Discussion

This case demonstrates the considerable difficulty that spinal TB produces in its diagnosis. This difficulty is especially true if one is not within an endemic setting. Our patient’s clinical course gives us an example of spinal TB disguised as a metastatic malignancy, both radiologically and clinically, resulting in a delay in diagnosis and unnecessary intervention, including radiotherapy in the absence of histopathological diagnosis.

The thoracic spine is the most commonly involved site for spinal TB. Most patients present with localized back pain. Neurological complications like sensory or motor weakness from growing compression of the spinal cord may follow [1,8]. But constitutional symptoms (e.g., fever, night sweats, or weight loss) are often absent, particularly in the immunocompetent host, further complicating the delay in recognition [15].

In this situation, vertebral body destruction along with lymphadenopathy and distant bony lesions raised a very strong suspicion of metastasis. This resulted in an extensive but ultimately misguided malignancy workup, involving significant imaging, PET scanning, and even a trial of radiotherapy, all of which were clinically ineffective. Research has shown that spinal TB can mimic a neoplastic process radiographically, which is due to lytic vertebra lesions, para-vertebral soft tissue involvement, and systemic spread [16,17]. Cases with similar presentations often report initial misdiagnosis as metastatic disease, demonstrating a recurring pattern of diagnostic mimicry in spinal TB. This emphasizes the need to consider TB in the differential, even when imaging strongly suggests malignancy.

TB can be distinguished from metastatic disease by the finding of caseating granulomas on histopathology, often with AFB. In the case of our patient, we made a definitive diagnosis after a CT-guided biopsy of the involved vertebral body. This demands that we emphasize the importance of tissue diagnosis in ambiguous spinal cases before the initiation of harmful radiation.

An additional factor in this case was the possibility that radiation-induced myelitis played a role in the patient’s ongoing sensory problems. Although radiation myelopathy is rare, it is a well-documented delayed complication of spinal radiotherapy, occurring months after treatment, and may present with progressive neurological symptoms that can be misinterpreted as disease progression or treatment failure [18]. It emphasizes the danger of commencing empirical treatment based on imaging alone when there is no diagnostic certainty.

Moreover, this case highlights the importance of considering TB in the differential diagnosis of spinal lesions in people from TB-endemic countries like Zimbabwe. A high index of suspicion should be kept even in the absence of typical systemic features or suggestive laboratory indicators. The diagnostic delay, despite multidisciplinary assessment and several imaging modalities, is consistent with the literature that reports a diagnostic delay of six months in cases of spinal TB [7].

When our patient started on appropriate ATT, she showed a significant clinical response, which is in keeping with the cure rate of 90% seen in patients who get treated promptly with standard regimens [14]. This patient showed marked recovery of neurological functions, suggesting that spinal cord compressions may be reversed if treatment is started before permanent damage occurs. Nonetheless, the irreversible consequences of irradiation, especially in non-malignant conditions, should act as a warning regarding premature therapeutic action.

This case reinforces the need for early biopsy in case of undifferentiated lesions in the spine. In a globalized world, clinicians based in "low-incidence" settings must remain vigilant of TB in migrants coming from endemic areas to avoid misdiagnosis and iatrogenic harm.

## Conclusions

The significance of a strong index of suspicion for spinal TB in cases of back pain and neurological deficits, especially in patients from endemic regions, is highlighted by this case. The unique imaging characteristics between spinal TB and cancer often confuse even experienced practitioners and can lead to unnecessary and potentially harmful treatments when tissue diagnosis is delayed. An early CT-guided biopsy is essential to differentiate between infectious and malignant causes. Timely initiation of the anti-tubercular treatment can ensure a dramatic clinical recovery, which can prevent any long-term neurological sequelae. This case also highlights the need for caution before administering empirical radiotherapy in the absence of histopathology.
